# A *Priestia megaterium* MF3 with high-efficiency zearalenone degradation: functional genomic insights and mechanistic exploration

**DOI:** 10.3389/fmicb.2025.1630165

**Published:** 2025-07-01

**Authors:** Di Meng, Kai-Zhong Xu, Hong-Jian Hou, Jin-Bin Liu, Dan-Dan Deng, Jun-Min Li, Ya-Kun Fang, Xiao-Qin Zhu, Dong-Li Pei

**Affiliations:** ^1^Henan Provincial Engineering Research Center for Development and Application of Characteristic Microorganism Resources, Shangqiu Normal University, Shangqiu, China; ^2^Key Laboratory of Tropical Biological Resources of Ministry of Education, School of Life and Health Sciences, Hainan University, Haikou, China; ^3^School of Marine and Bioengineering, YanCheng Institute of Technology, Yancheng, Jiangsu, China

**Keywords:** zearalenone, *Priestia megaterium*, biodegradation, enzyme, whole-genome sequence

## Abstract

Zearalenone (ZEN), a mycotoxin produced by *Fusarium* species, is widely distributed and poses significant health risks to both animals and humans due to its toxic effects. In this study, a *Priestia megaterium* MF3, exhibiting high ZEN degradation capacity, was identified through comprehensive morphological, physicochemical, 16S rRNA gene sequencing, and whole-genome sequencing analyses. Strain MF3 reached its peak ZEN degradation rate in BHI medium (pH 7, 30°C), with > 90% efficiency maintained across 24–72 h, 1–5% inoculum, and 10–40 μg/mL ZEN. The ZEN-degrading activity of strain MF3 was attributed to both extracellular and intracellular components, with extracellular enzymes in the fermentation supernatant playing a predominant role. LC-MS analysis identified key ZEN degradation products, including 1-(3,5-dihydroxyphenyl)-6’-hydroxy-1’-undecen-10’-one, ZEN-P, and zearalanone. Whole-genome sequencing further revealed the presence of genes encoding α/β hydrolases and phosphotransferases, which are likely involved in the hydrolysis and phosphorylation of ZEN. Furthermore, strain MF3 demonstrated an impressive ability to remove 81.78% of ZEN from moldy corn within 12 h. This study not only identifies a highly efficient bacterial strain for ZEN biodegradation but also provides valuable insights into its degradation mechanism, offering potential applications for mycotoxin detoxification in the food and feed industries.

## 1 Introduction

Zearalenone (ZEN), a secondary metabolite with the chemical structure 6-(10-hydroxy-6-oxo-trans-1-undecenyl)-β-resorcylic acid lactone, is predominantly produced by *Fusarium* species (Altomare et al., 2021). It is commonly found in contaminated food and feed products, including corn, barley, wheat, and other grains, as well as their derivatives (Xu et al., 2021; Ji et al., 2023). ZEN has been shown to exert hepatotoxic, immunotoxic, and genotoxic effects (Zinedine et al., 2007; Wang et al., 2018). Additionally, it has been classified as a Group 3 carcinogen by the International Agency for Research on Cancer (Murtaza et al., 2024). Regulatory authorities worldwide have implemented rigorous standards controlling ZEN concentrations, as defined by established maximum residue thresholds. EU legislation sets varying ZEN limits for different cereal products: 100 μg/kg for raw cereals (excluding maize), 75 μg/kg for cereals meant for direct human consumption, and 20 μg/kg for processed cereal foods, particularly emphasizing baby food products (André et al., 2024). The Chinese National Food Safety Standard establishes a 60 μg/kg maximum residue limit for ZEN in cereals and their processed derivatives (Cai et al., 2024). Consequently, the swift and efficient removal of ZEN residues has become a critical concern that demands immediate attention in the field of food and feed safety.

At present, physical, chemical, and biological methods are commonly utilized for the removal of ZEN (Yu et al., 2012; Fang et al., 2022; Murtaza et al., 2024). Biological methods, including microbial adsorption, microbial degradation, and enzymatic degradation, have become the most efficient due to their high effectiveness, low cost, and environmentally friendliness (Król et al., 2018; Xu et al., 2022). To date, numerous microorganisms capable of efficiently eliminating ZEN have been successfully isolated (Cai et al., 2024; Gari, 2024). Among the bacteria capable of eliminating ZEN, *Priestia* is a newly identified genus that was reclassified from *Bacillus* (Gupta et al., 2020). Similar to *Bacillus*, it is considered the most promising candidate due to its moderate nutritional needs, production of various bioactive compounds, and ability to sporulate, which allows it to survive under harsh environmental conditions, including extreme temperatures and pH levels (Stein, 2005; Hassan et al., 2021). For instance, *Bacillus megaterium* BM344-1 (now known as *Priestia megaterium*) was reported to degrade 25% of 1.5 μg/mL of ZEN in maize within 44 h (Hassan et al., 2021). *Bacillus pumilus* ANSB01G was found to degrade 88.65, 84.58, 83.04, and 66.34% of ZEN in liquid culture medium, natural moldy corn, pig feed, and distiller’s grains, respectively (Lei et al., 2014). In a similar vein, *B. amyloliquefaciens* ZDS-1 demonstrated high efficiency in degrading ZEN at high concentrations (1 mg/L to 100 mg/L) (Xu et al., 2016). Additionally, enzymatic activity is crucial in microbial detoxification, facilitating the transformation of mycotoxins into harmless or less harmful metabolites (Fadia et al., 2019; Sun et al., 2020). For example, the lactonohydrolase ZHD101 derived from *Gliocladium roseum* has been identified to convert ZEN into 1-(3,5-dihydroxyphenyl)-6’-hydroxy-1’-undecen-10’-one, a metabolite devoid of estrogenic activity (Takahashi-Ando et al., 2002). An intracellular zearalenone phosphotransferase from *B. subtilis* Y816 was able to convert ZEN into ZEN-14-phosphate, which exhibited reduced estrogenic toxicity compared to ZEN (Yang et al., 2021). Although microorganisms have demonstrated great potential in the ZEN degradation, their degradation efficiency is often limited by prolonged degradation period, underscoring the need to screen strains with high degradation capacity. Furthermore, the underlying mechanisms of degradation by numerous strains have not been fully elucidated, and the potential toxicity of their metabolic byproducts remains unassessed, which significantly restricts their practical applicability (Liu et al., 2023).

Therefore, the aims of this study were to (1) isolate and characterize a strain *Priestia megaterium* MF3 with high ZEN degradation capacity; (2) optimize its ZEN degradation conditions, including culture medium, culture time, inoculation size, pH, temperature, and ZEN concentrations; (3) assess the effects of its active components on ZEN degradation; (4) analyze and identify the ZEN degradation products; (5) analyze its genome to identify potential genes involved in ZEN degradation; and (6) evaluate its ZEN degradation ability in moldy corn.

## 2 Materials and methods

### 2.1 Reagents and cultivation medium

Zearalenone (ZEN) was obtained from Sigma-Aldrich (St. Louis, Missouri, United States). Sodium dodecyl sulfate (SDS) and proteinase K were sourced from Sangon Biotech (Shanghai, China), while ethylenediaminetetraacetic acid (EDTA) was acquired from Macklin (Shanghai, China). Corn was purchased from a local farmers’ market in Shangqiu City. Other chemical reagents, unless otherwise stated, were all analytical grade.

Mineral salt medium (MSM) used for the enrichment culture of soil samples consisted of Na_2_HPO_4_ (6.8 g/L), KH_2_PO_4_ (3 g/L), NaCl (0.5 g/L), NH_4_Cl (1 g/L), MgSO_4_ (0.24 g/L), and CaCl_2_ (0.011 g/L). Luria-Bertani broth (LB), Nutrient broth (NB), Tryptic soy broth (TSB), and Brain heart infusion (BHI) broth were employed for strain culturing and their components are detailed in Supplementary Table S1.

### 2.2 Screening, isolation, and purification of zearalenone-degrading bacteria

Soil samples were collected from Dongzhaigang mangrove reserve (19°57′N, 110°34′E), Hainan, China. This region (3,337.3 hm^2^, including 1,576.24 hm^2^ of mangrove) has a typical tropical maritime climate, with annual precipitation ranging from 1,600 to 2,000 mm and a mean annual temperature of 23.8°C (Hu et al., 2015; Zhang et al., 2024). The area experiences irregular diurnal tides with an average tidal range of approximately 1 m (Zhang et al., 2024). As China’s most pristine and biodiverse mangrove wetland, it hosts 35 mangrove species, accounting for 95% of the country’s total. The predominant soil type is acid sulfate soil with deep nearshore soil layer and abundant organic matter (Hu et al., 2015). For enrichment, the soil sample was introduced into MSM medium supplemented with 10 μg/mL ZEN at a 2% (w/v) ratio, followed by incubation at 37°C and 180 rpm for 24 h. The culture solutions were then sampled, serially diluted, and spread onto MSM solid agar (containing 10 μg/mL of ZEN), followed by another 24–h incubation at 37°C. Individual colonies were aseptically selected and further streaked onto LB agar plate until uniform single colonies were obtained. The preliminary isolates were then inoculated into LB medium containing 10 μg/mL ZEN and cultured at 37°C and 180 rpm for 24 h. LB medium with 10 μg/mL of ZEN, incubated under identical conditions, was used as the control.

ZEN quantification was performed using LC-MS/MS analysis (Qtrap™ 6500^+^, SCIEX, United States), where 5 μL aliquots were introduced into the system operating in negative ion electrospray ionization (ESI) mode. The LC-MS/MS conditions were set as follows: Kinetex^®^ C18 column (2.1 × 150 mm, particle 1.7 μm, Phenomenex, United States); mobile phase A: water with 0.1% formic acid and 5% acetonitrile; mobile phase B: 95% acetonitrile with 0.1% formic acid; column temperature: 40°C; flow rate: 0.3 mL/min; gradient program: 0–5 min, 25–70% B; 5–6 min, 70% B; 6–6.1 min, 70–25%B; 6.1–8 min, 25%B. MS/MS parameters were as follows: ionization mode: negative ESI; ion spray voltage: −4500 V; source temperature: 350°C; nebulizer gas (GS1): 30 psi; drying gas (GS2): 6 L/min; curtain gas: 30 psi; MRM (Multiple Reaction Monitoring) mode was used to detect ZEN; parent ion (Q1): m/z 317.1 → product ion (Q3): m/z 175; declustering potential (DP): −60 V; collision energy: −24 eV; dwell time: 100 ms. ZEN concentration was quantified using the external standard method, based on a calibration curve constructed from serial dilutions of ZEN standards. The linearity of the calibration curve (R^2^ > 0.99) was used to calculate the sample concentration based on peak area. This approach has been previously applied and validated in ZEN detection by LC-MS/MS (Burak and Buket, 2023). The ZEN degradation rate was calculated using the formula:


$$\mathrm{Degradation}\mathrm{rate}\left(\%\right)=\left(1-\frac{A_{1}}{A_{0}}\right)\times 100$$

where *A*_0_ and *A*_1_ represented the peak area of ZEN in the control and experimental groups, respectively.

### 2.3 Identification of strain MF3

Strain MF3 was identified through morphological observation, Gram staining, physiological and biochemical assays (Guerrero, 2001), and 16S rRNA sequence analysis. Physiological and biochemical characterization were conducted using HBI biochemical identification system (Hope Bio-Technology Co., Ltd., Qingdao, China), assessing citrate utilization, propionate metabolism, *D*-xylose and *D*-arabinose assimilation, *D*-Mannitol fermentation, Voges-Proskauer (V-P) reaction, gelatin liquefaction, tolerance to 7% NaCl and pH 5.7, nitrate reduction, and starch hydrolysis. The genomic DNA of MF3 strain was extracted following the MiniBEST Bacteria Genomic DNA Extraction Kit (TaKaRa, DaLian, China). The 16S rRNA gene fragment was amplified using the universal primers 27F (5′-AGAGTTTGATCCTGGCTCAG-3′) and 1492R (5′-GGTTACCTTGTTACGACTT-3′) (Liu et al., 2023; Wang et al., 2024), and the resulting PCR product was subsequently sequenced by Tsingke Biotech (Beijing, China). Sequence alignment was performed using the NCBI Blast Tool for homology analysis (Altschul et al., 1990). A phylogenetic tree of strain MF3 was generated using the Neighbor-Joining method in MEGA 11 software (Tamura et al., 2021).

### 2.4 The growth and ZEN degradation of strain MF3 in different culture media

Strain MF3 was incubated at 37°C and 180 rpm for 12 h. The overnight culture solution of strain MF3 was then centrifuged at 8,000 × *g* and 37°C for 5 min. The harvested cells were resuspended in the sterile saline to an optical density equivalent to 0.5 McFarland standard. Cell suspensions were inoculated (1% of inoculation, v/v) into different media (LB, NB, TSB, and BHI medium) containing ZEN at a final concentration of 10 μg/mL. Control groups either contained the same concentration of ZEN but were not inoculated with bacterial cells or were inoculated with bacterial cells but without the addition of ZEN. The media were then incubated at 37°C and 180 rpm. For ZEN degradation analysis, samples were collected after 24 h of fermentation. Samples were taken at 0, 2, 4, 6, 8, 12, 16, 20, 24, 28, 32, 36, and 40 h to analyze the growth strain MF3 in different media containing 10 μg/mL of ZEN.

### 2.5 Effect of culture conditions on ZEN degradation by strain MF3

0.5 McFarland standard of cell suspension was obtained as described in section 2.4 and used for subsequent determination the effect of culture conditions (culture time, inoculum size, initial pH, temperature, and ZEN concentration) on ZEN degradation by strain MF3.

For the culture duration experiment, a 1% (v/v) inoculum of cell suspension was introduced into BHI medium supplemented with ZEN (final concentration of 10 μg/mL and incubated) and then cultured at the condition of 37°C and 180 rpm. Samples were collected at designated time points (6, 12, 24, 48, and 72 h) for further analysis.

For the inoculum size test, cell suspension was inoculated into BHI medium (containing 10 μg/mL of ZEN) with different inoculum size (1, 2, 3, 4, and 5%; v/v) and cultured at 37°C, 180 rpm for 24 h.

For the initial pH test, cell suspension was inoculated (5% of inoculation, v/v) into BHI medium (containing 10 μg/mL of ZEN) with pH values of 3, 4, 5, 6, 7, 8, and 9. The mixture was incubated at 37°C and 180 rpm for 24 h.

In the temperature experiment, a 5% (v/v) cell suspension was added to BHI medium containing 10 μg/mL of ZEN, and the mixture was incubated at different temperatures (20°, 25°, 30°, 37°, and 42°C) with agitation at 180 rpm for 24 h.

For the ZEN concentration assay, a 5% (v/v) inoculum was introduced into BHI medium supplemented with different initial ZEN concentrations (1.25, 2.5, 5, 10, 15, 20, and 40 μg/mL) and cultured at 30°C with shaking at 180 rpm for 24 h.

Control groups contained the same concentration of ZEN but without bacteria inoculation, were subjected to identical incubation conditions as the experimental groups. The cell density of strain MF3 was assessed by measuring absorbance at 600 nm.

### 2.6 ZEN degradation by different components of strain MF3 culture broth

A cell suspension with a turbidity of 0.5 McFarland standard was inoculated into BHI medium containing 10 μg/mL ZEN at a 5% (v/v) ratio and cultured at 30°C with shaking at 180 rpm for 12 h. Cells and fermentation supernatants were separated by centrifugation (8,000 *× g*, 4°C, 15 min). The collected cells were washed three times with an equal volume of phosphate-buffered saline (PBS, pH 7.2, 20 mM) and resuspended in an equivalent volume of PBS. The resuspended cells were disrupted via ultrasonication (600 W, 15 min) in an ice bath using intermittent pulses (5 s on 5 s off) to prevent overheating. The resulting mixture was then centrifuged at 10,000 *× g* and 4°C for 15 min to collect the cell disruption supernatant. Heat inactivated cells were prepared by heating resuspended cells at 100°C for 15 min. Each component (bacterial solution, viable cells, heat inactivated cells, cell disruption supernatants, and fermentation supernatants, respectively) was supplemented with ZEN to a final concentration of 10 μg/mL and incubated at 30°C with shaking at 180 rpm for 12 h. For the bacterial solution and fermentation supernatant tests, BHI medium containing with 10 μg/mL of ZEN served as the control. PBS (pH 7.2, 20 mM) containing 10 μg/mL of ZEN was used as the control for the viable cells, cell disruption supernatant, and heat inactivated cell groups.

### 2.7 ZEN degradation by strain MF3 fermentation supernatant with different treatment

The fermentation supernatant of strain MF3 was prepared as described in section 2.6. The fermentation supernatant was subjected to various treatments, including incubation with protease K (200 μg/mL) at 37°C for 1 h, heating at 100°C for 15 min, and treatment with SDS (10%, w/v) or EDTA (5 mM) at 37°C for 30 min, respectively. The fermentation supernatant without protease K, heating, SDS, or EDTA served as the control. All samples were supplemented with ZEN to a final concentration of 10 μg/mL and incubated at 30°C with shaking at 180 rpm for 12 h.

### 2.8 Analysis of degradation products of ZEN by strain MF3

Strain MF3 was cultured in BHI medium under the optimal conditions described above. Samples were prepared according to Liu et al. (2023) and analyzed by LCMS-IT-TOF (Shimadzu, Tokyo, Japan) using the method of Xu et al. (2024). The mobile phase A and B were the same as these mentioned in section 2.2. The gradient program was set as follows: 0–1 min, 10% B; 1–7 min, 10–100% B; 7–9 min, 100%B; 9–9.01 min, 100–10%B; 9.01–10 min, 10%B. The injection volume was 3 μL. The flow rate was 0.3 mL/min. The electrospray ion source operated in positive ion mode, with a scan range of 100−500 *m/z*. The interface voltage was set to 3.5 kV. Nitrogen was used as the nebulizing gas at a flow rate of 1.5 L/min and a pressure of 200 kPa. The curved desolvation line (CDL) and the heat block were both maintained at 200°C. The ion accumulation time was 30 ms, and the precursor ion selection width was set to 3.0 amu. Argon served as both the cooling and collision gas, with the collision-induced dissociation (CID) energy set at 50%. The pressure in the time-of-flight (TOF) region was maintained at 1.5 × 10^–4^ Pa, while that in the ion trap was 1.7 × 10^–2^ Pa. The detector voltage was set to 1.64 kV.

### 2.9 ZEN degradation in moldy corn by strain MF3

The ZEN degradation test for moldy corn was performed using the method described by Hu et al. (2023), with minor modifications. Specifically, the reaction mixture consisted of 1 g of moldy corn flour, 3.5 mL of PBS (pH 7.2, 20 mM), and 0.5 mL of strain MF3 bacterial solution. The reaction mixture was incubated at 37°C with shaking at 180 rpm for 12 h. Following incubation, samples were collected by centrifugation (10,000 *× g*, 4°C, 15 min). The control group involved using a heat-inactivated MF3 bacterial solution. All the samples were subsequently analyzed using LCMS-IT-TOF (Shimadzu, Tokyo, Japan), as described in section 2.8.

### 2.10 Genome sequencing and bioinformatics analysis

Genomic analysis of strain MF3 was performed by Majorbio (Shanghai, China). Sequencing was performed using a combination of PacBio Sequel II and Illumina platforms. The raw Illumina sequencing reads were screened using Fastp (v0.20.0) to obtain clean short reads. The HiFi reads were generated from the PacBio platform. The obtained clean short reads and HiFi reads were assembled to construct complete genomes using Flye (v2.9.2) (Kolmogorov et al., 2019). To reduce the rate of small errors, Pilon (v1.22) was used to polish the assembly using short-read alignments (Walker et al., 2014). Finally, a gap-free circular genome was constructed successfully. The genome annotation was carried out using the NCBI Prokaryotic Genome Annotation Pipeline. The 16S rRNA sequence (accession number: PV262373) and the whole genome sequence data (accession number: chromosome, CP185256; plasmid A, CP185257; plasmid B, CP185258; and plasmid C, CP185259) have been archived in the NCBI database.

### 2.11 Statistical analysis

Each experiment was repeated three times. Statistical analysis was performed using one-way ANOVA followed by Tukey’s *post-hoc* test for multiple group comparisons. For pairwise comparisons between related samples, a two-tailed paired *t*-test was applied.

## 3 Results and discussion

### 3.1 Isolation and identification of ZEN-degrading strain MF3

Following the initial plate screening and subsequent flask screening, the exceptional ZEN-degrading ability of strain MF3 was demonstrated following 24 h of incubation in LB medium with 10 μg/mL of ZEN. During this incubation, new metabolic compounds were identified (Supplementary Figure S1).

**FIGURE 1:**
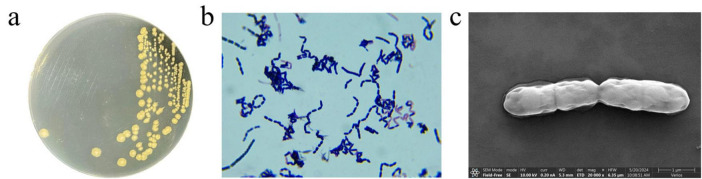
Morphological characteristics of strain MF3. **(a)** Colony morphology of strain MF3 on LB agar, **(b)** Gram-staining of strain MF3 cells observed under a microscope (1000 × magnification), **(c)** Scanning electron micrographs of strain MF3.

To identify strain MF3, its morphological, physic-biochemical, and molecular characteristics were analyzed. As shown in Figure 1, strain MF3 was identified as a rod-shaped, Gram-positive bacterium, and its colonies are flat, round or oval, opaque, and white, transitioning to light yellow upon growth. Similar to most *Bacillus* Spp., strain MF3 exhibited the ability to hydrolyze gelatin and starch (Cui et al., 2023). In the HBI biochemical identification system test, strain MF3 tested positive for the V-P test and the utilization of L-arabinose and D-mannitol but tested negative for citrate, propionate, and D-xylose. In addition, strain MF3, which lacked nitrate reduction capability, could thrive under pH 5.7 conditions and tolerate up to 7% NaCl (Table 1). Phylogenetic analysis based on the 16S rRNA gene and housekeeping genes, along with subsequent whole-genome comparisons (Supplementary Figure S2), strain MF3 was found to be closely related to *Priestia megaterium*, a species previously classified under the genus *Bacillus* (Gupta et al., 2020). Moreover, the average nucleotide identity (ANI) value of MF3 and *Priestia megaterium* (GCF 009497655.1) was 99.42% (Supplementary Figure S2d), indicating that strain MF3 may belong to the same species as *Priestia megaterium* (GCF 009497655.1) (Li et al., 2024). Nevertheless, significant differences were observed in the physiological and biochemical characteristics (Table 1), particularly in the V-P tests, where strain MF3 tested positive while *P. megaterium* yielded negative results (Cui et al., 2023; Hossain et al., 2021; Liu et al., 2010; Pinki et al., 2021; Wagh et al., 2021).

**TABLE 1:** Physiological and biochemical characteristics of strain MF3 and other *Priestia megaterium* species.

Characteristics	1	2	3	4	5	6	7	8
V-P test	+	−	−	−	−	−	−	−
Citrate	−	ND	ND	ND	ND	+	+	−
Propionate	−	ND	ND	ND	ND	ND	−	ND
D-xylose	−	ND	ND	ND	ND	ND	ND	−
L-arabinose	+	ND	ND	ND	ND	ND	ND	−
D-mannitol	+	ND	ND	ND	ND	ND	ND	ND
Gelatin liquefaction	+	+	+	+	+	ND	ND	ND
7% NaCl	+	ND	ND	ND	ND	ND	+	−
pH 5.7 growth	+	ND	ND	ND	ND	ND	+	ND
Nitrate reduction	−	ND	ND	ND	ND	+	+	−
Starch hydrolysis	+	+	+	+	+	ND	+	ND

Strain: 1, MF3 (this study); 2, *Priestia megaterium* TGB1 (Hossain et al., 2021), 3, 4, and 5, *Priestia megaterium* KD5, *Priestia megaterium* KD7, and *Priestia megaterium* KD8 (Cui et al., 2023); 6, *Priestia megaterium* E7 (Pinki et al., 2021), 7, *Priestia megaterium* (Liu et al., 2010); 8, *Priestia megaterium* IAM 13418^T^ (Wagh et al., 2021).+, positive, −, negative; ND, not determined.

### 3.2 The growth and ZEN degradation of strain MF3 in different culture media

**FIGURE 2:**
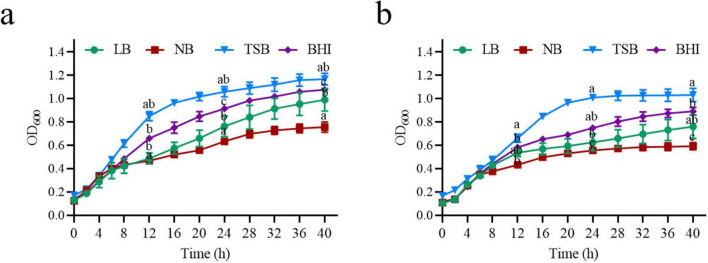
Growth curve of strain MF3 in different culture media. **(a)** Growth curve of strain MF3 in different culture media without ZEN, **(b)** Growth curve of strain MF3 in different culture media with 10 μg/mL of ZEN. Significant differences between media at each time point were determined using one-way ANOVA with Tukey’s *post-hoc* test (*p* < 0.05). Different lowercase letters indicate significant differences between media.

**FIGURE 3:**
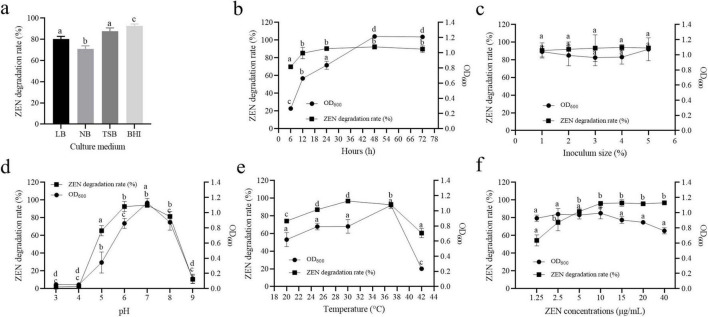
Effect of the culture conditions on the growth and ZEN degradation of strain MF3. **(a)** Culture medium, **(b)** culture time, **(c)** inoculum size, **(d)** pH, **(e)** temperature, and **(f)** ZEN concentration. Data are presented as mean ± SD (*n* = 3). Statistical significance among groups was determined by Tukey’s *post-hoc* test (*p* < 0.05). Different lowercase letters indicate significant differences between different groups.

The composition of the culture medium is crucial for microbial growth and the bio-decomposition of pollutants (Zhai et al., 2022; Gari, 2024). To evaluate the growth and ZEN degradation of strain MF3, four common media (LB, NB, TSB, and BHI) were tested. The growth curves of strain MF3 in these media showed similar trends, both with and without ZEN supplementation. Although the presence of ZEN slightly reduced biomass production, it had minimal impact on the growth of strain MF3 (Figure 2). The findings align with those of Ju et al. (2019), who reported that ZEN exhibited minimal effects on the growth of *B. natto*. When comparing the growth and ZEN degradation of strain MF3 across different culture media, strain MF3 exhibited better growth and the highest ZEN degradation in BHI medium, followed by TSB medium, where it also showed robust growth and significant ZEN degradation. In contrast, strain MF3 grew the slowest and degraded the least ZEN in NB medium (Figures 2b, 3a).

### 3.3 Effect of culture conditions on ZEN degradation by strain MF3

Microbial growth and pollutant degradation are influenced by various factors, including culture time, inoculation volume, temperature, pH, and ZEN concentration (Yang et al., 2022; Liu et al., 2023). To evaluate their impact on ZEN degradation by strain MF3, these factors were systematically tested. As shown in Figure 3b, strain MF3 successfully degraded ZEN at all tested incubation times. The ZEN degradation rate by strain MF3 increased from 0 to 24 h and then stabilized from 24 to 72 h. In accordance with our findings, Yang et al. (2022) stated that the ZEN degradation rates of *Proteus mirabilis* SY-3, *B. subtilis* SY-14, and *B. subtilis* SY-20 all stabilized after a certain period. However, strain MF3 exhibited rapid growth during the initial 48 h, followed by a stable phase. Interestingly, the peak degradation rate occurred before MF3 reached its growth stable phase. This suggested that the number of cells and the quantity of secreted substances were sufficient for ZEN degradation during the exponential phase.

Inoculation size also influenced ZEN degradation. A previous study by Yang et al. (2022) reported no significant difference in ZEN degradation by *B. subtilis* SY-14 when the inoculum size ranged from 1 to 9%, although the 1% inoculum size resulted in a higher degradation rate. Similarly, for strain MF3, no significant differences in ZEN degradation or growth were observed across various inoculum sizes. However, a 5% inoculum size yielded the highest growth and ZEN degradation rate (Figure 3c).

The pH of the culture medium is one of the most critical factors influencing microbial growth and enzymatic reactions (Hornbaek et al., 2004; Liu et al., 2023; Murtaza et al., 2024). Here, an experiment was conducted to evaluate the effects of different initial pH values on the growth and ZEN degradation of strain MF3. As shown in Figure 3d, strain MF3 was capable of growth and efficient ZEN degradation within a pH range of 5.0–8.0, with the optimal growth and ZEN degradation observed at pH 7.0. Extreme pH values (e.g., 3, 4, and 9) inhibited both growth and ZEN degradation. Similar studies have also reported that *B. spizizenii* B73, another ZEN-degrading strain, exhibited optimal growth and degradation activity at pH 7.0 (Liu et al., 2023).

Temperature is a critical factor influencing microbial growth and metabolic activity (Murtaza et al., 2024; Wang et al., 2024). Strain MF3 demonstrated efficient ZEN degradation across all tested temperatures ranges (Figure 3e). The degradation rate of ZEN increased with temperature up to 30°C, after which it gradually decreased, reaching 60.57% at 42°C. Thus, strain MF3 exhibited optimal ZEN degradation at 30°C. This finding is consistent with Xu et al. (2016), who reported that the optimal temperature for ZEN degradation by *B. amyloliquefaciens* ZDS-1 was 30°C, but its ZEN degradation rate decreased rapidly to less than 10% at 40°C. Additionally, strain MF3 was able to grow within a temperature range of 20–42°C, with optimal growth occurring at 37°C. Interestingly, while growth activity decreased at 42°C, strain MF3 still maintained a higher ZEN degradation rate, suggesting that the enzymes involved in ZEN degradation have a broad temperature tolerance.

ZEN is recognized for its capacity to disrupt both DNA replication and protein biosynthesis, which may consequently affect microbial proliferation and cellular integrity (Wang et al., 2020; Liu et al., 2023). Therefore, this study investigated the effects of ZEN concentration on the growth and degradation capabilities of strain MF3. As shown in Figure 3f, ZEN degradation was positively correlated with ZEN concentration in the range of 1.25–10 μg/mL. ZEN degradation rates exhibited no significant difference within the range of 10–40 μg/mL, with all rates exceeding 95%. Strain MF3 exhibited robust growth across ZEN concentration of 1.25–40 μg/mL, although growth gradually declined at concentrations above 10 μg/mL. This is likely due to the toxicity of higher ZEN concentrations, which may impair cell function and inhibit strain growth (Yang et al., 2021).

### 3.4 ZEN degradation by different components of strain MF3 culture broth

**FIGURE 4:**
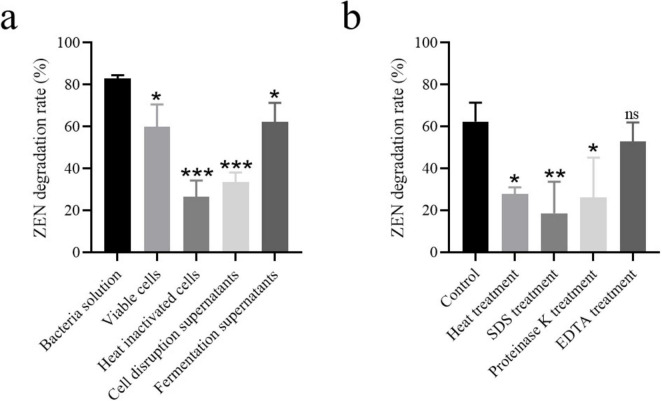
**(a)** ZEN degradation by different components of strain MF3 culture broth, **(b)** ZEN degradation by strain MF3 fermentation supernatant with heat, SDS, proteinase K, or EDTA treatment. Statistical significance among groups was determined by Tukey’s *post-hoc* test. *P < 0.05, **P < 0.01, ***P < 0.001.

Absorption and degradation are the primary mechanisms for microbial removal of ZEN (Xu et al., 2016; Yang et al., 2022; Murtaza et al., 2024). To investigate the mechanism of ZEN removal by strain MF3, the ZEN degradation capabilities of different components were assessed, including bacterial solution, viable cells, heat inactivated cells, cell disruption supernatants, and fermentation supernatants. As shown in Figure 4a, fermentation supernatants and viable cells exhibited higher ZEN degradation rates of 62.36 and 60.07%, respectively. Cell disruption supernatants demonstrated a moderate ZEN degradation rate of 33.44%. In addition, heat inactivated cells still showed 26.48% of ZEN degradation rate. It can be concluded that both cell adsorption and degradation contribute to ZEN removal, with the ZEN degradation by strain MF3 primarily originating from extracellular supernatant and intracellular substances (Murtaza et al., 2024).

### 3.5 ZEN degradation by strain MF3 fermentation supernatant with different treatment

To determine whether ZEN degradation was enzymatic, the fermentation supernatants were treated with protease K, heat, adding SDS or EDTA prior to exposure to ZEN. As shown in Figure 4b, the ZEN degradation ability of the fermentation supernatants significantly decreased after all treatments compared to the untreated control. SDS treatment resulted in the lowest ZEN degradation rate (18.58%) in the fermentation supernatants. ZEN degradation rates of the fermentation supernatants treated with protease K or heat were 26.19 and 27.77%, respectively. Furthermore, the ZEN degradation rate of the fermentation supernatants decreased to 52.96% after treatment with EDTA. Protein activity is significantly compromised through multiple mechanisms: SDS-induced structural denaturation, enzymatic degradation by protease K, thermal destabilization, and EDTA’s metal ion sequestration, all of which ultimately impair ZEN degradation capacity (Bhattacharyya et al., 1994; Zhang et al., 2020; Hu et al., 2023). Based on our results, enzymes in the fermentation supernatants of strain MF3 likely contribute to ZEN degradation, and the activity of some enzymes involved in the ZEN degradation process may depend on the presence of metal ions.

### 3.6 Characterization of ZEN degradation products by strain MF3

To investigate the mechanism underlying ZEN degradation by strain MF3, degradation intermediates were analyzed using an LCMS-IT-TOF system. ZEN exhibited a retention time (RT) of 6.96 min, with its mass spectrum showing a molecular ion at *m/z* 319 [M+H]^+^ (Supplementary Figure S3). The RTs of three new products, identified as P1, P2, and P3, were 0.84 min, 4.52 min, and 5.18 min, respectively (Supplementary Figures S4–S6).

**FIGURE 5:**
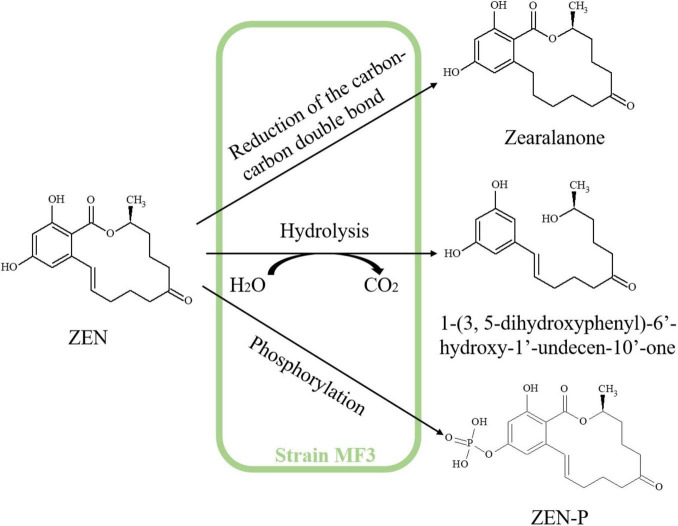
Proposed ZEN degradation pathway of strain MF3.

According to the mass spectrum characteristics of P1 (Supplementary Figure S4), the molecular ion peak at *m/z* 321 [M+H]^+^ (2 Da higher than that of ZEN) suggests that P1 is likely zearalanone, produced by the reduction of C1’ = C2’double bond in ZEN, as previously reported by Cai et al. (2024). The protonated ion of P2 was detected at *m/z* 293, corresponding to a molecular weight of 292, with its mass spectrum matching the molecular formula C_17_H_24_O_4_ (Supplementary Figure S5). 1-(3,5-dihydroxyphenyl)-6’-hydroxy-1’-undecen-10’-one, with the chemical formula C_17_H_24_O_4_, is a known intermediate of ZEN hydrolysis, as reported by Liu et al. (2023) and Cai et al. (2024). Furthermore, ZEN exhibits a typical macrolide structure, and the hydrolysis of its lactone ring is a well-established degradation pathway (Wang et al., 2017). Therefore, P2 was identified as 1-(3,5-dihydroxyphenyl)-6’-hydroxy-1’-undecen-10’-one. For P3, the protonated ion at *m/z* 399 and its MS spectrum was consistent with ZEN-P (Supplementary Figure S6; Yang et al., 2021). Additionally, *B. subtilis* Y816, which belongs to the same family as strain MF3, has been reported to transform ZEN into its phosphorylated conjugate, ZEN-P (Yang et al., 2021). Therefore, P3 was inferred as ZEN-P. The structures of ZEN degradation products are summarized in Table 2. Based on the identified metabolites, the ZEN degradation by strain MF3 possibly involved three metabolic pathways: reduction of the carbon-carbon double bond, hydrolysis, and phosphorylation (Figure 5). In addition to elucidating the metabolic pathways of ZEN degradation, future research should also focus on the toxicological assessment of the resulting metabolites. In particular, the estrogenic activities of zearalanone (P1), ZEN-P (P3), and other transformation products warrant further investigation, for instance, using *in vitro* assays such as the MCF-7 cell proliferation model. Such evaluations are essential to ensure the biosafety and practical applicability of strain MF3 in food and feed detoxification.

**TABLE 2:** Identification of the ZEN degradation products by strain MF3.

Products	RT (min)	Measured mass [M + H]^+^ (m/z)	Common name	Proposed structure	References
P1	0.84	321	Zearalanone	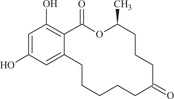	(Cai et al., 2024)
P2	4.52	293	1-(3, 5-dihydroxyphenyl)-6’-hydroxy-1’-undecen-10’-one	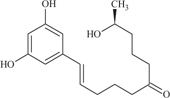	(Liu et al., 2023; Cai et al., 2024)
P3	5.18	399	ZEN-P	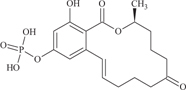	(Yang et al., 2021)

### 3.7 ZEN degradation in moldy corn by strain MF3

**FIGURE 6:**
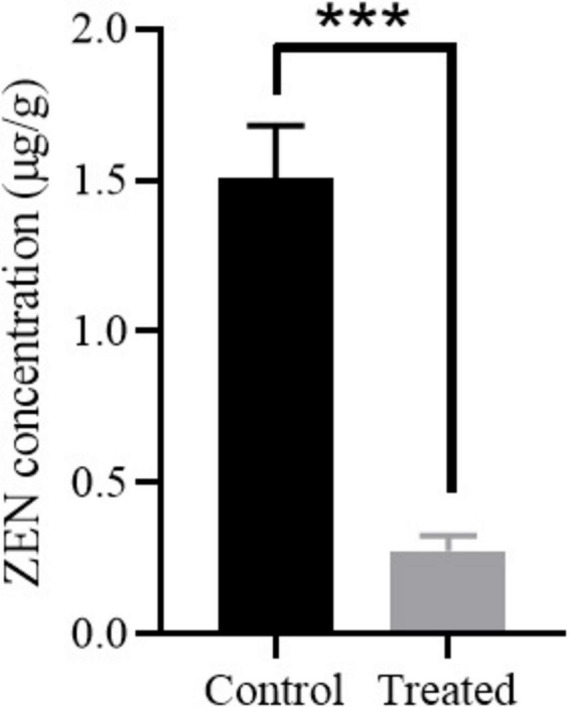
ZEN degradation in moldy corn by strain MF3. The control group and treated group were the corn treated with heat-inactivated strain MF3 and active strain MF3, respectively. Statistically significant differences were marked with *** (*P* < 0.001) using Tukey’s test.

Corn, one of the most common foods susceptible to ZEN contaminations, was selected to evaluate the ZEN degradation efficiency of strain MF3. As shown in Figure 6, the ZEN concentration in moldy corn was significantly reduced from 1.51 to 0.27 μg/g after 12 h of treatment with strain MF3, falling below the regulatory threshold of 0.5 μg/g for corn in feed (Ropejko and Twarużek, 2021; Hu et al., 2023). In comparison, Ju et al. (2019) reported *B. subtilis* achieved 75% ZEN degradation in corn flour after 48 h. Guo et al. (2020) demonstrated that *B. velezensis* ANSB01E removed 75.36% of ZEN from moldy corn meal after 48 h, while *B. spizizenii* B73 reduced ZEN concentration in corn meal by 80.31% after 36 h (Liu et al., 2023). Notably, in the present study, strain MF3 achieved a significant ZEN reduction of 81.78% for moldy corn after 12 h, highlighting its potential as a feed additive for mitigating ZEN contamination in feed.

### 3.8 Genome sequencing and bioinformatics analysis

**FIGURE 7:**
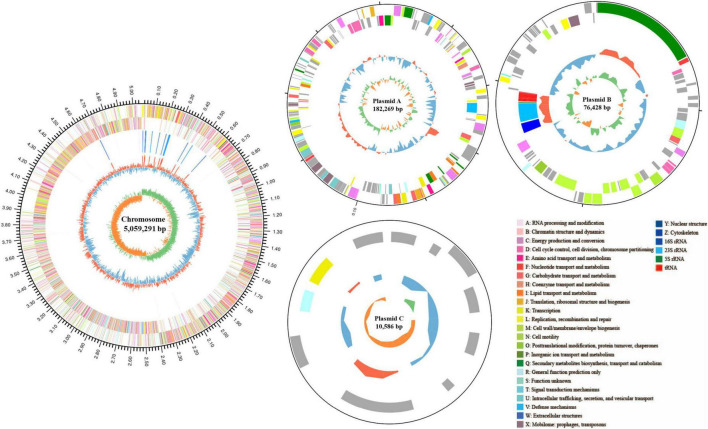
Schematic representation of the complete *Priestia* sp. MF3 genome. The circles, numbered from the outermost (first) to the innermost (sixth), represent the following features: the scale line (first circle), with each major tick marking 0.1 Mb, coding DNA sequences on both the forward and reverse strands, with distinct colors indicating clusters of orthologous groups of proteins (COGs) categories (second and third circles); rRNA and tRNA (fourth circle); guanine-cytosine (GC) content (fifth circle); and GC skew (sixth circle). The circular diagram was created using Circos 0.69.6 (http://www.circos.ca).

To investigate the genomic characteristics, identify ZEN degradation-related genes, and explore the degradation mechanism of strain MF3, its entire genome was sequenced. The genome of strain MF3 consists of 1 chromosome and 3 plasmid (Figure 7). The total genome size of strain MF3 is 5,328,574 bp, with an average GC content of 38.06%. The genome of strain MF3 contains 5,382 coding sequences (CDS), 125 tRNA genes, and 42 rRNA genes. These genomic features are similar to those of other complete *Priestia* genus genomes deposited in the NCBI database, with genome sizes ranging from 3.817 to 6.873 Mb, GC contents from 36.5 to 61.5%, and CDS numbers from 3,740 to 6,963 (Supplementary Table S2). The 5,382 CDS were annotated using several databases, including NR, Swiss-Prot, Pfam, COG, GO, and KEGG. The number of genes assigned to each database were 5,374, 4,095, 4,421, 4,051, 2,940, and 3,679, respectively.

In the present study, hydrolysis, phosphorylation, and reduction of the carbon-carbon double bond occurred during the ZEN degradation process by strain MF3, yielding 1-(3,5-dihydroxyphenyl)-6’-hydroxy-1’-undecen-10’-one, ZEN-P, and zearalanone. Therefore, A BLAST comparison of known ZEN-degrading enzymes was performed with the genome of strain MF3. Seven genes (gene1670, gene1672, gene2399, gene3401, gene3653, gene4846, and gene4906) were annotated as encoding α/β hydrolase and exhibited 24.4, 30.5, 21.4, 22.1, 30.9, 20.7, and 26.9% amino acid identity, respectively, with the well-known ZEN hydrolase ZHD101, which hydrolyzes ZEN into 1-(3,5-dihydroxyphenyl)-10’-hydroxy-1’-undecen-6’-one (Xiang et al., 2016; Hu et al., 2023). Compared to ZHD101, these seven genes all belonged to the α/β hydrolase family and contained the characteristic α/β hydrolase domain, as further confirmed by phylogenetic analysis (Supplementary Table S3; Supplementary Figure S7a).

BLAST analysis further revealed that gene1321, gene1641, gene1828, and gene4732, functionally annotated as phosphoenolpyruvate-protein phosphotransferase, PEP/pyruvate-binding domain-containing protein, phosphoenolpyruvate synthase, and pyruvate kinase, respectively, exhibited 34.1, 49.4, 27.8, and 31.5% amino acid identity with ZEN phosphotransferase (MZ170042.1), which has been reported to convert ZEN into ZEN-14-phosphate (Yang et al., 2021). Notably, gene1641 not only clustered in the same phylogenetic branch as ZEN phosphotransferase (MZ170042.1), but also shared the most conserved domain with it (Supplementary Figures S7b, S8).

Pompa et al. (1988) investigated the *in vitro* transformation of α/β-ZAL and zearalanone (ZAN) by oxo-reductases in microsomal and cytosolic fractions obtained from lamb livers, demonstrating that ZAN production depended on the presence of NAD/NADH as a co-factor. Numerous genes in the strain MF3 have been annotated as NAD(P) or NAD(P)H-dependent oxidoreductase (data not shown). Notably, only one gene, gene3254, encoding a bifunctional cytochrome P450/NADPH-P450 reductase, was identified in the strain MF3 genome (Supplementary Table S3). However, to the best of our knowledge, the specific enzyme responsible for converting ZEN to zearalanone has not been reported, necessitating further research to identify enzymes involved in this metabolic process.

## 4 Conclusion

In this study, a *Priestia megaterium* MF3, exhibiting high ZEN degradation capacity, was identified through comprehensive morphological, physicochemical, 16S rRNA gene sequencing, and whole-genome sequencing analyses. ZEN degradation capacity of strain MF3 was enhanced by optimizing culture conditions, including culture composition, culture time, inoculation size, pH, temperature, and ZEN concentration. The active components responsible for ZEN degradation were found both extracellularly and intracellularly, with enzymatic activity in the extracellular fermentation supernatant playing a key role in ZEN degradation. LC-MS analysis identified the key ZEN degradation products as 1-(3,5-dihydroxyphenyl)-6’-hydroxy-1’-undecen-10’-one, ZEN-P, and zearalanone. Strain MF3 significantly reduced the ZEN concentration in moldy corn. Additionally, certain genes in the MF3 genome were annotated to encode α/β hydrolase and phosphotransferase, which may contribute to the hydrolysis and phosphorylation of ZEN. Further studies are needed to identify the specific enzymes involved in the conversion of ZEN to zearalanone.

## Data Availability

The datasets presented in this study can be found in online repositories. The names of the repository/repositories and accession number(s) can be found in Supplementary material.
