# Supplementary material for: A Priestia megaterium MF3 with high-efficiency zearalenone degradation: functional genomic insights and mechanistic exploration
Source: Front Microbiol. 2025 Jul 1;16:1630165. doi: 10.3389/fmicb.2025.1630165 (PMC12259703; doi:10.3389/fmicb.2025.1630165)
Supplement: Supplementary file 1 [file Data_Sheet_1.docx]

**Supplementary materials**


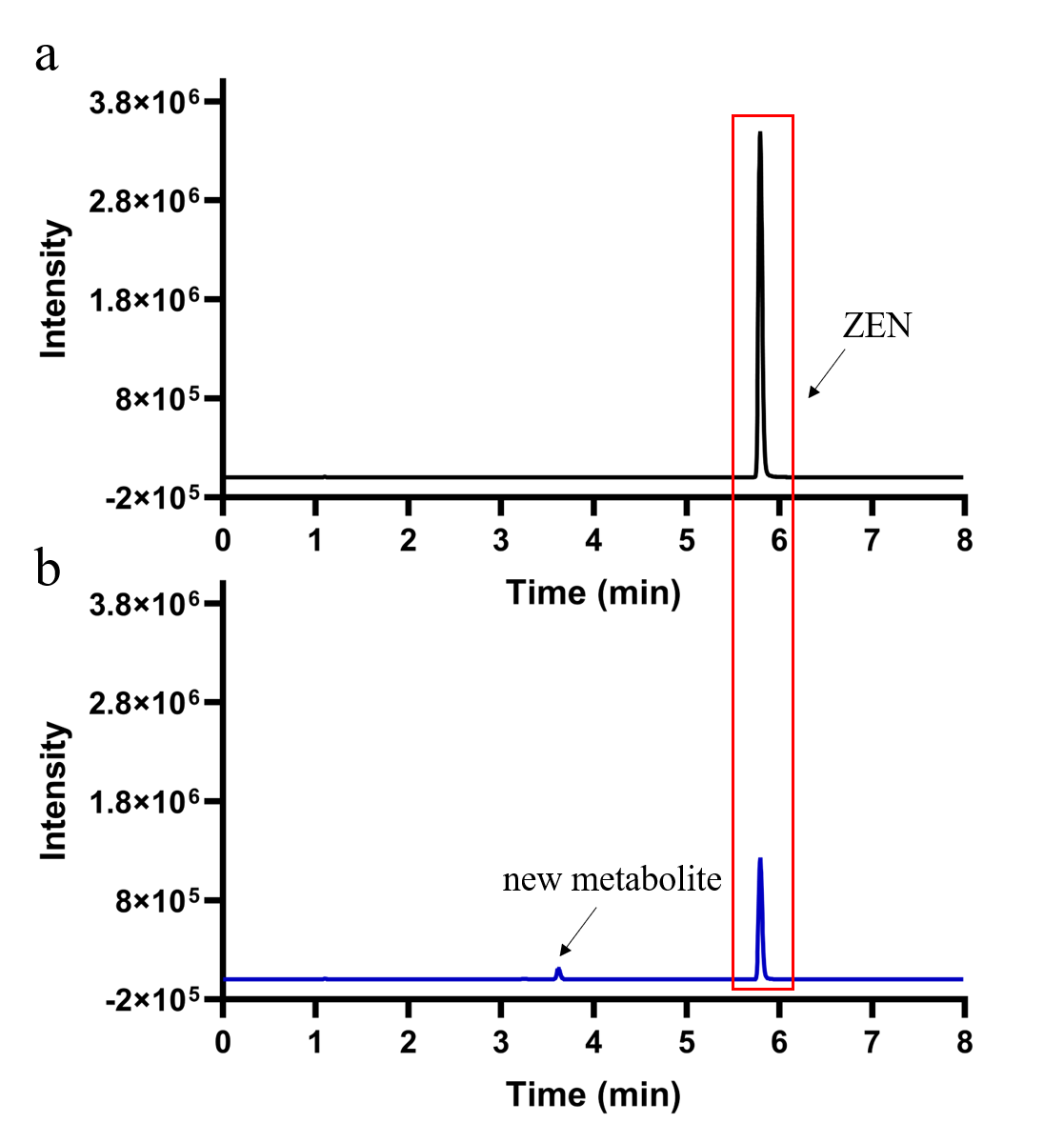


FIGURE S1 LC-MS/MS profile of ZEN degradation by strain MF3. (a) LB medium with 10 µg/mL ZEN; (b) a culture of strain MF3 grow on LB medium with 10 µg/mL ZEN for 24 h.


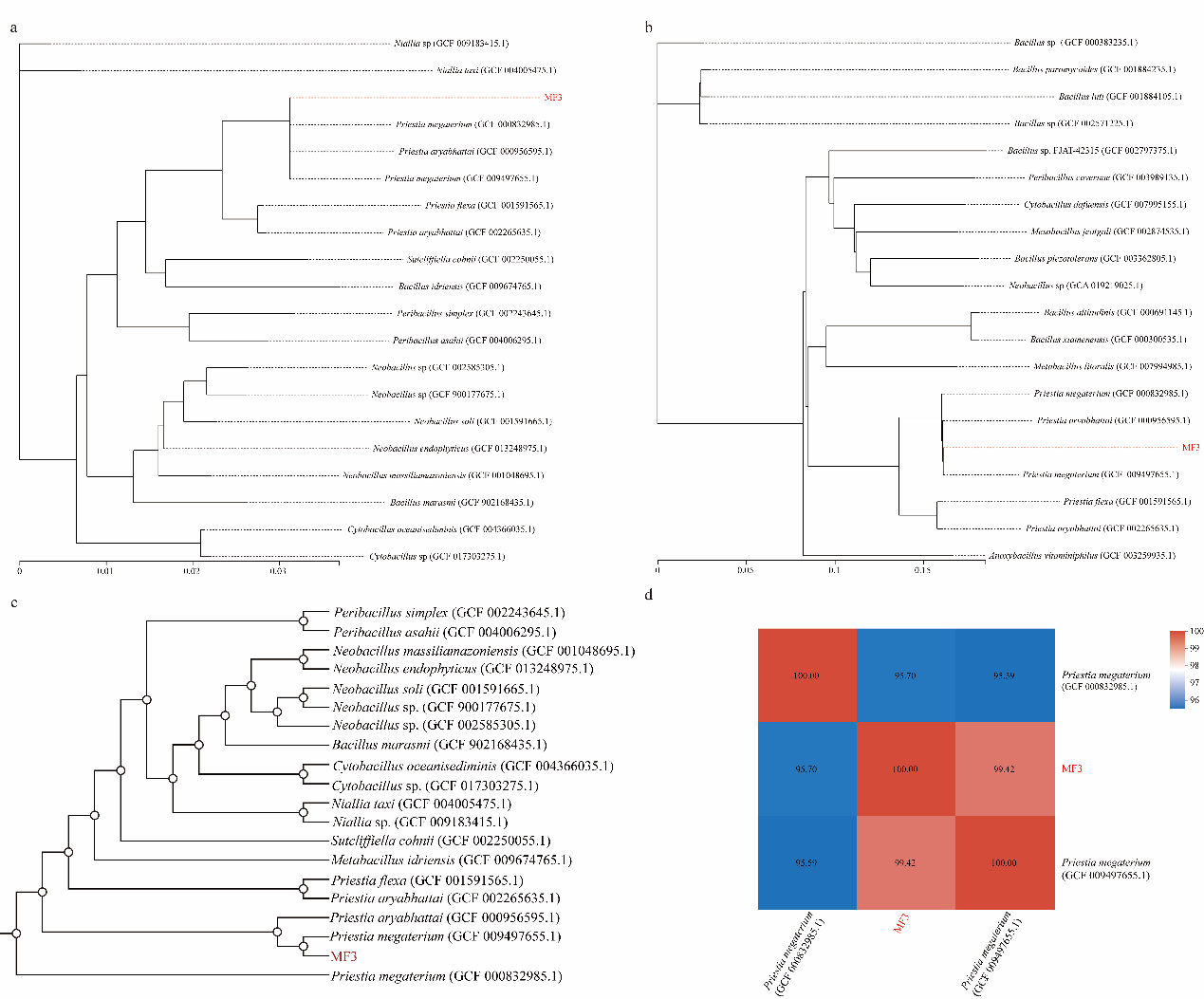


FIGURE S2 Taxonomic identification of strain MF3. (a) The phylogenetic tree based on 16S rRNA sequences; (b) The phylogenetic tree based on housekeeping genes (*dnaG*, *frr*, *infC*, *nusA*, *pgk*, *pyrG*, *rplA*, *rplB*, *rplC*, *rplD*, *rplE*, *rplF*, *rplK*, *rplL*, *rplM*, *rplN*, *rplP*, *rplS*, *rplT*, *rpmA*, *rpoB*, *rpsB*, *rpsC*, *rpsE*, *rpsI*, *rpsJ*, *rpsK*, *rpsM*, *rpsS*, *smpB*, *tsf*); (c) The phylogenetic tree based on genome sequences of strain MF3 and other strains downloaded from NCBI; (d) Average nucleotide identity (ANI) values (%) among MF3, *Priestia megaterium* (GCF 000832985.1), and *Priestia megaterium* (GCF 009497655.1).

The phylogenetic trees based on 16S rRNA sequences and housekeeping genes were constructed using the Neighbor-Joining (NJ) algorithm in MEGA 6.0 software. Whole-genome sequence tree was generated by WebCVTree v4. ANI analysis was performed on the online tool of Majorbio Cloud Platform (https://cloud.majorbio.com/page/tools/).


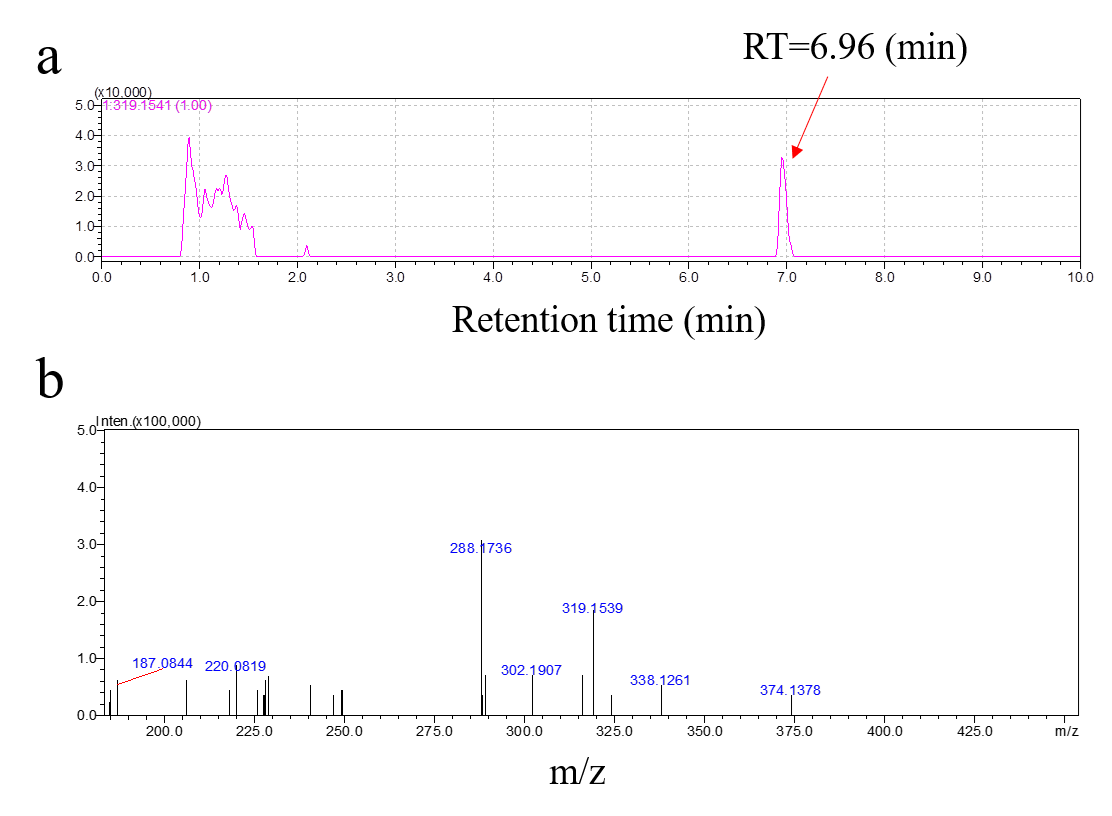


FIGURE S3 Liquid chromatogram (a) and mass spectra (b) of ZEN.


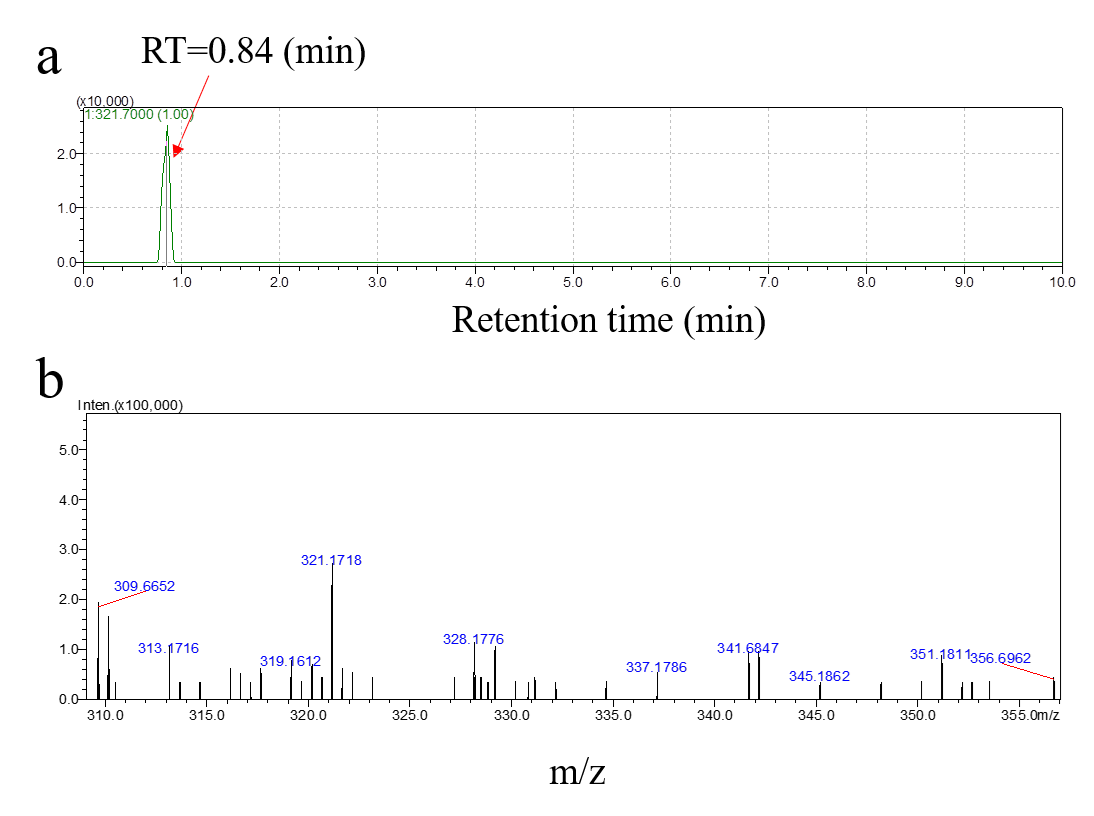


FIGURE S4 Liquid chromatogram (a) and mass spectra (b) of P1.


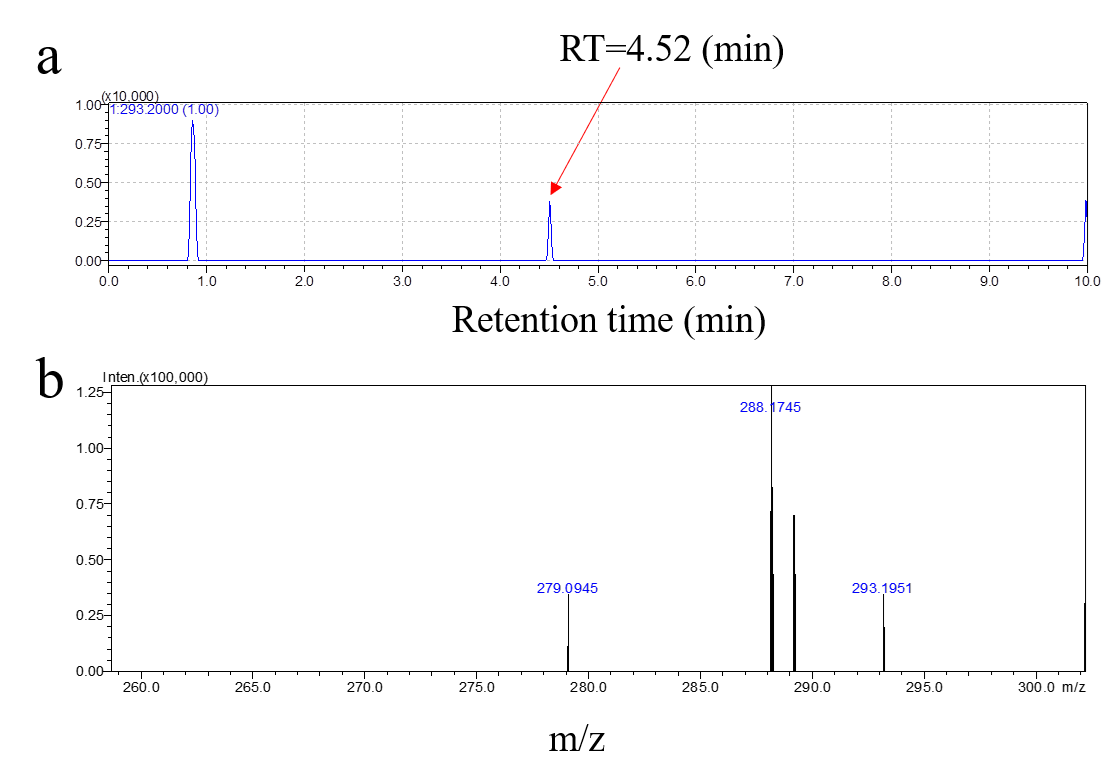


FIGURE S5 Liquid chromatogram (a) and mass spectra (b) of P2.


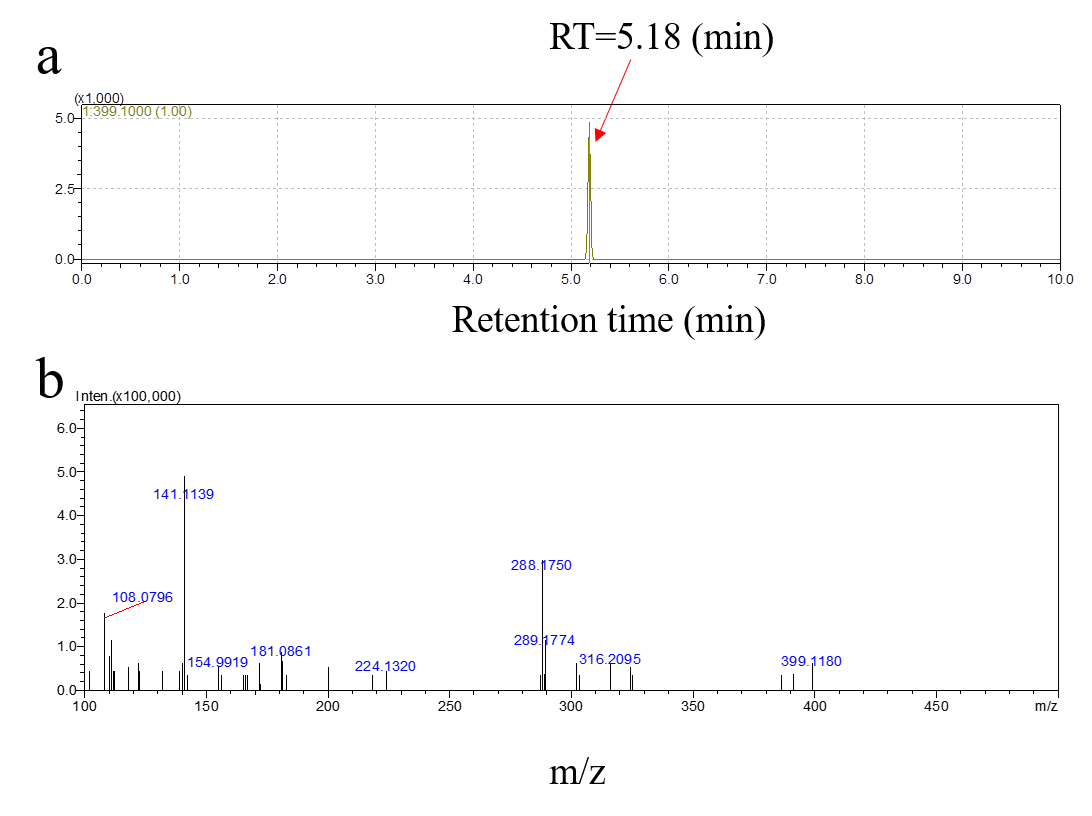


FIGURE S6 Liquid chromatogram (a) and mass spectra (b) of P3.


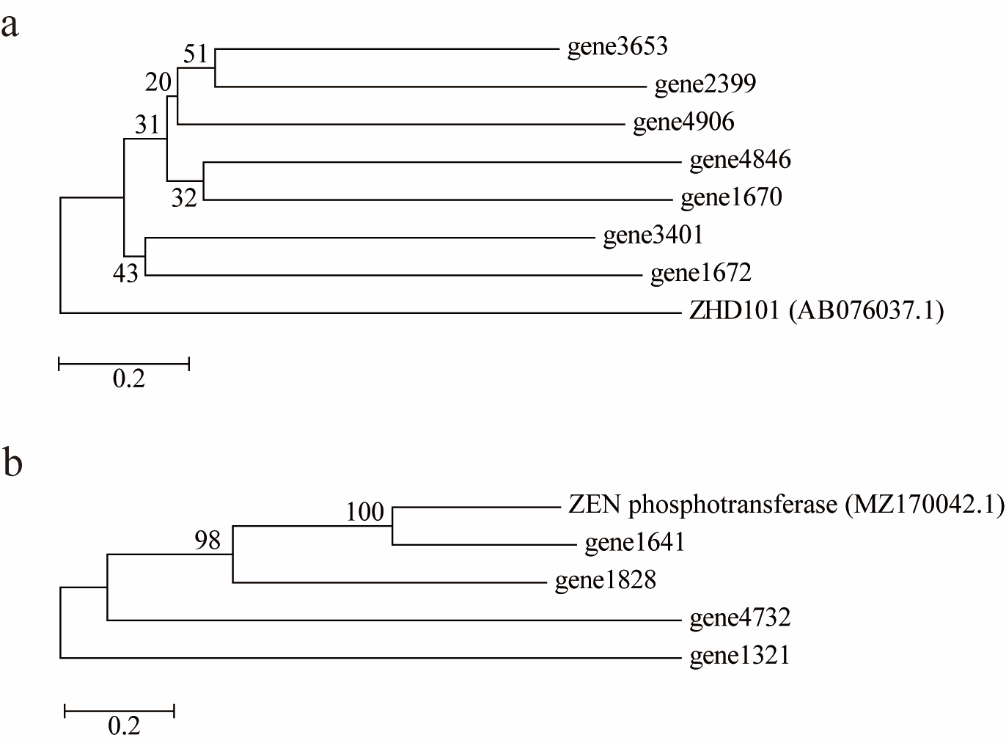


FIGURE S7 Phylogenetic trees based on CDSs of potential ZEN-degrading genes. (a) The phylogenetic tree based on CDSs of potential ZEN-degrading genes and ZHD101 (AB076037.1); (b) The phylogenetic tree based on CDSs of potential ZEN-degrading genes and ZEN phosphotransferase (MZ170042.1). This tree was constructed by the neighbor-joining (NJ) algorithm and bootstrap values were based on 1000 replications. It showed the phylogenetic relationship between reference queries and their genes by BLAST comparison.


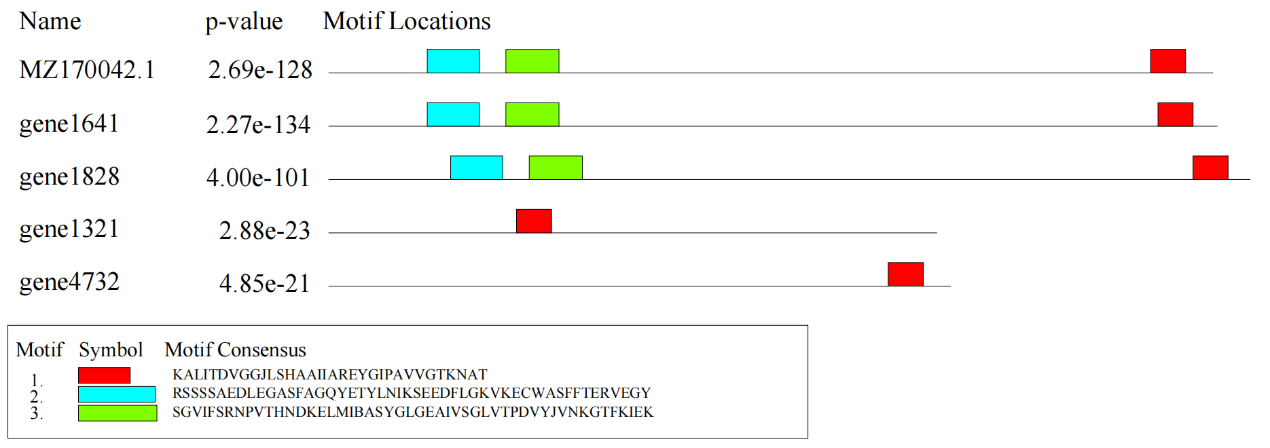


FIGURE S8 Motif locations analysis. It was generated by MEME Suite 5.5.7.

TABLE S1 Culture medium used in this study.

| Medium type | Component | Concentration (g/L) |
| --- | --- | --- |
| Luria-Bertani broth (LB) medium | yeast extract | 5 |
|  | peptone | 10 |
|  | NaCl | 10 |
| Nutrient broth (NB) medium | beef extract powder | 3 |
|  | peptone | 10 |
|  | NaCl | 5 |
| Tryptic soy broth (TSB) medium | tryptone | 17 |
|  | soybean papain hydrolysate | 3 |
|  | glucose | 2.5 |
|  | K_2_HPO_4_ | 2.5 |
|  | NaCl | 5 |
| Brain heart infusion (BHI) medium | peptone | 10 |
|  | dehydrated calf brain soaked powder | 12.5 |
|  | dehydrated beef heart soaked powder | 5 |
|  | NaCl | 5 |
|  | glucose | 2 |
|  | Na_2_HPO_4_ | 2.5 |

TABLE S2 Complete genome summary of ***Priestia*** groups

| Scientific name | GenBank | Level | Genome size (Mb) | GC content (%) | Protein-coding genes (CDS) |
| --- | --- | --- | --- | --- | --- |
| *Priestia aryabhattai* | GCA_002688605.1 | Complete | 5.254 | 38 | 5,265 |
| *Priestia aryabhattai* | GCA_007106705.1 | Complete | 5.487 | 38 | 5,416 |
| *Priestia aryabhattai* | GCA_015832345.1 | Complete | 6.216 | 61.5 | 5,659 |
| *Priestia aryabhattai* | GCA_017743055.1 | Complete | 5.957 | 37.5 | 5,986 |
| *Priestia aryabhattai* | GCA_019968665.1 | Complete | 5.258 | 38 | 5,246 |
| *Priestia aryabhattai* | GCA_022811825.1 | Complete | 5.450 | 38 | 5,445 |
| *Priestia aryabhattai* | GCA_028751835.1 | Complete | 5.087 | 38 | 5,046 |
| *Priestia aryabhattai* | GCA_028891465.1 | Complete | 5.213 | 38 | 5,167 |
| *Priestia aryabhattai* | GCA_028994175.1 | Complete | 5.507 | 38 | 5,496 |
| *Priestia aryabhattai* | GCA_030374065.1 | Complete | 5.318 | 38 | 5,308 |
| *Priestia aryabhattai* | GCA_030406185.1 | Complete | 5.734 | 38 | 5,714 |
| *Priestia aryabhattai* | GCA_030486505.1 | Complete | 5.295 | 38 | 5,334 |
| *Priestia aryabhattai* | GCA_036785815.1 | Complete | 5.052 | 38 | 5,055 |
| *Priestia aryabhattai* | GCA_037081845.1 | Complete | 5.296 | 38 | 5,310 |
| *Priestia aryabhattai* | GCA_041545735.1 | Complete | 5.571 | 38 | 5,545 |
| *Priestia aryabhattai* | GCA_041902395.1 | Complete | 5.943 | 37.5 | 5,961 |
| *Priestia aryabhattai* | GCA_043522765.1 | Complete | 5.317 | 38 | 5,305 |
| *Priestia filamentosa* | GCA_035678045.2 | Complete | 4.574 | 36.5 | 4,413 |
| *Priestia filamentosa* | GCA_000972245.3 | Complete | 5.314 | 36.5 | 5,210 |
| *Priestia filamentosa* | GCA_028471965.1 | Complete | 4.263 | 37 | 4,138 |
| *Priestia flexa* | GCA_002024265.1 | Complete | 4.104 | 38 | 3,971 |
| *Priestia flexa* | GCA_005577315.1 | Complete | 4.019 | 38 | 3,968 |
| *Priestia flexa* | GCA_021441905.1 | Complete | 4.000 | 38 | 3,932 |
| *Priestia flexa* | GCA_029536875.1 | Complete | 3.994 | 38 | 3,938 |
| *Priestia flexa* | GCA_030122985.1 | Complete | 3.817 | 38 | 3,740 |
| *Priestia flexa* | GCA_040212095.1 | Complete | 4.081 | 38 | 4,041 |
| *Priestia koreensis* | GCA_022646885.1 | Complete | 4.758 | 40 | 4,746 |
| *Priestia megaterium* | GCA_040364115.1 | Complete | 5.170 | 38 | 5,217 |
| *Priestia megaterium* | GCA_001648135.1 | Complete | 5.037 | 38.5 | 5,046 |
| *Priestia megaterium* | GCA_002009195.1 | Complete | 5.611 | 38 | 5,545 |
| *Priestia megaterium* | GCA_003072605.2 | Complete | 5.432 | 38 | 5,393 |
| *Priestia megaterium* | GCA_003351965.1 | Complete | 5.509 | 38 | 5,569 |
| *Priestia megaterium* | GCA_006385935.1 | Complete | 5.713 | 38 | 5,686 |
| *Priestia megaterium* | GCA_009497655.1 | Complete | 5.243 | 38 | 5,278 |
| *Priestia megaterium* | GCA_009911775.1 | Complete | 5.172 | 38.5 | 5,189 |
| *Priestia megaterium* | GCA_011058275.1 | Complete | 5.407 | 38 | 5,387 |
| *Priestia megaterium* | GCA_012275205.1 | Complete | 6.469 | 38.5 | 5,952 |
| *Priestia megaterium* | GCA_013146705.1 | Complete | 6.873 | 37 | 6,963 |
| *Priestia megaterium* | GCA_013389435.1 | Complete | 5.985 | 37.5 | 5,965 |
| *Priestia megaterium* | GCA_013458535.1 | Complete | 6.165 | 37.5 | 6,145 |
| *Priestia megaterium* | GCA_015582655.1 | Complete | 5.374 | 38 | 5,455 |
| *Priestia megaterium* | GCA_015643545.1 | Complete | 5.148 | 38 | 5,155 |
| *Priestia megaterium* | GCA_017086525.1 | Complete | 5.748 | 38 | 5,752 |
| *Priestia megaterium* | GCA_017086545.1 | Complete | 5.609 | 38 | 5,547 |
| *Priestia megaterium* | GCA_017086565.1 | Complete | 6.000 | 38 | 5,956 |
| *Priestia megaterium* | GCA_017352315.1 | Complete | 6.412 | 37.5 | 6,446 |
| *Priestia megaterium* | GCA_017798265.1 | Complete | 5.269 | 38 | 5,252 |
| *Priestia megaterium* | GCA_022023815.1 | Complete | 5.923 | 37.5 | 5,869 |
| *Priestia megaterium* | GCA_022537925.1 | Complete | 5.605 | 38 | 5,585 |
| *Priestia megaterium* | GCA_022811865.1 | Complete | 5.428 | 38 | 5,410 |
| *Priestia megaterium* | GCA_022811885.1 | Complete | 5.303 | 38 | 5,338 |
| *Priestia megaterium* | GCA_022870665.1 | Complete | 5.257 | 38 | 5,217 |
| *Priestia megaterium* | GCA_023824135.1 | Complete | 6.101 | 37.5 | 6,041 |
| *Priestia megaterium* | GCA_023824155.1 | Complete | 5.684 | 38 | 5,652 |
| *Priestia megaterium* | GCA_023824175.1 | Complete | 6.162 | 37.5 | 6,101 |
| *Priestia megaterium* | GCA_023824195.1 | Complete | 5.984 | 37.5 | 5,982 |
| *Priestia megaterium* | GCA_025913555.1 | Complete | 5.304 | 38 | 5,315 |
| *Priestia megaterium* | GCA_025919585.1 | Complete | 5.250 | 38 | 5,240 |
| *Priestia megaterium* | GCA_028622975.1 | Complete | 5.310 | 38 | 5,366 |
| *Priestia megaterium* | GCA_028751625.1 | Complete | 5.510 | 38 | 5,355 |
| *Priestia megaterium* | GCA_029536995.1 | Complete | 5.698 | 38 | 5,648 |
| *Priestia megaterium* | GCA_029537115.1 | Complete | 5.313 | 38 | 5,257 |
| *Priestia megaterium* | GCA_029873355.1 | Complete | 5.086 | 38 | 5,065 |
| *Priestia megaterium* | GCA_030291715.1 | Complete | 5.488 | 38 | 5,486 |
| *Priestia megaterium* | GCA_030520145.1 | Complete | 5.298 | 38 | 5,252 |
| *Priestia megaterium* | GCA_033450805.1 | Complete | 6.004 | 37.5 | 5,997 |
| *Priestia megaterium* | GCA_034479695.1 | Complete | 5.703 | 38 | 5,663 |
| *Priestia megaterium* | GCA_037039575.1 | Complete | 6.156 | 37.5 | 6,125 |
| *Priestia megaterium* | GCA_038447705.1 | Complete | 5.777 | 37.5 | 5,767 |
| *Priestia megaterium* | GCA_040114905.1 | Complete | 5.507 | 38 | 5,543 |
| *Priestia megaterium* | GCA_041941685.1 | Complete | 6.000 | 37.5 | 5,878 |
| *Priestia megaterium* | GCA_042691625.1 | Complete | 5.751 | 37.5 | 5,733 |
| *Priestia megaterium* | GCA_045278405.1 | Complete | 5.093 | 38.5 | 5,084 |
| *Priestia megaterium* | GCA_045348665.1 | Complete | 5.294 | 38 | 5,316 |
| *Priestia megaterium* | GCA_045819105.1 | Complete | 5.611 | 38 | 5,609 |
| *Priestia megaterium* DSM 319 | GCA_000025805.1 | Complete | 5.097 | 38 | 5,057 |
| *Priestia megaterium* DSM 319 | GCA_029537015.1 | Complete | 5.103 | 38 | 4,999 |
| *Priestia megaterium* NBRC 15308 = ATCC 14581 | GCA_000832985.1 | Complete | 5.747 | 38 | 5,745 |
| *Priestia megaterium* NBRC 15308 = ATCC 14581 | GCA_006094495.1 | Complete | 5.747 | 38 | 5,754 |
| *Priestia megaterium* NCT-2 | GCA_000334875.3 | Complete | 5.884 | 38 | 5,802 |
| *Priestia megaterium* Q3 | GCA_001050455.1 | Complete | 5.23 | 38 | 5,250 |
| *Priestia megaterium* QM B1551 | GCA_000025825.1 | Complete | 5.523 | 38 | 5,497 |
| *Priestia megaterium* WSH-002 | GCA_000225265.1 | Complete | 5.075 | 38 | 5,049 |
| *Priestia* sp. HNGD-A6 | GCA_045784335.1 | Complete | 5.321 | 38 | 5,341 |
| *Priestia* sp. J2 | GCA_020790095.1 | Complete | 5.314 | 38 | 5,321 |
